# The New Jersey Institute of Technology Robot-Assisted Virtual Rehabilitation (NJIT-RAVR) system for children with cerebral palsy: a feasibility study

**DOI:** 10.1186/1743-0003-6-40

**Published:** 2009-11-16

**Authors:** Qinyin Qiu, Diego A Ramirez, Soha Saleh, Gerard G Fluet, Heta D Parikh, Donna Kelly, Sergei V Adamovich

**Affiliations:** 1 New Jersey Institute of Technology, Department of Biomedical Engineering, University Heights Newark, NJ 07102, USA; 2 University of Medicine and Dentistry of New Jersey, Department of Rehabilitation and Movement Science, 65 Bergen Street Newark, NJ 07107, USA; 3 Children's Specialized Hospital 150 New Providence Road, Mountainside, NJ 07092, USA

## Abstract

**Background:**

We hypothesize that the integration of virtual reality (VR) with robot assisted rehabilitation could be successful if applied to children with hemiparetic CP. The combined benefits of increased attention provided by VR and the larger training stimulus afforded by adaptive robotics may increase the beneficial effects of these two approaches synergistically. This paper will describe the NJIT-RAVR system, which combines adaptive robotics with complex VR simulations for the rehabilitation of upper extremity impairments and function in children with CP and examine the feasibility of this system in the context of a two subject training study.

**Methods:**

The NJIT-RAVR system consists of the Haptic Master, a 6 degrees of freedom, admittance controlled robot and a suite of rehabilitation simulations that provide adaptive algorithms for the Haptic Master, allowing the user to interact with rich virtual environments. Two children, a ten year old boy and a seven year old girl, both with spastic hemiplegia secondary to Cerebral Palsy were recruited from the outpatient center of a comprehensive pediatric rehabilitation facility. Subjects performed a battery of clinical testing and kinematic measurements of reaching collected by the NJIT-RAVR system. Subjects trained with the NJIT-RAVR System for one hour, 3 days a week for three weeks. The subjects played a combination of four or five simulations depending on their therapeutic goals, tolerances and preferences. Games were modified to increase difficulty in order to challenge the subjects as their performance improved. The testing battery was repeated following the training period.

**Results:**

Both participants completed 9 hours of training in 3 weeks. No untoward events occurred and no adverse responses to treatment or complaints of cyber sickness were reported. One participant showed improvements in overall performance on the functional aspects of the testing battery. The second subject made improvements in upper extremity active range of motion and in kinematic measures of reaching movements.

**Conclusion:**

We feel that this study establishes the feasibility of integrating robotics and rich virtual environments to address functional limitations and decreased motor performance in children with mild to moderate cerebral palsy.

## Introduction

Cerebral palsy (CP) is a non progressive neurodevelopmental disorder of motor control due to lesions or other dysfunctions of the CNS [1]. Every 2 to 3 out of 1000 newborn babies are diagnosed with cerebral palsy [1]. Cerebral palsy produces motor dysfunction and depending on lesion location, deficits in sensation, sensorimotor processing, and coordinated movements in multiple muscle groups [2]. Hemiplegia occurs in approximately one third of diagnosed CP cases and consists of disturbances in tone and movement of the involved side. The involved upper extremity significantly impacts play and self-care activities such as eating and dressing [3].

Current motor learning theory describes a correlation between improved motor function and the use of "massed" or "repetitive" practice [4]. Constraint induced movement therapy (CIMT) is currently being used in children to accomplish the goals of intensive massed practice and shaping. It has demonstrated the ability to produce sustained improvement in motor function in children with spastic hemiplegia secondary to CP [5,6]. Multiple authors describe improvements maintained at six month retention [7]. High levels of attention and motivation are required for this type of training to be successful [7], which can limit its feasibility for some children. Other novel approaches to rehabilitation of children with hemiplegia include a bilateral focused approach to manual intervention which includes the use of both upper extremities in intensive training without the use of a constraint. Gordon et al describes a brief (10 day) program of massed practice utilizing both hands to improve bilateral upper extremity function in children with cerebral palsy [8].

Virtual reality (VR) is another technology used to accomplish intensive massed practice in children. VR therapy has the capability to create an interactive, motivating environment in which the therapist can manipulate the practice intensity and feedback to create individualized treatments [9]. Use of VR is thought to enhance children's motivation, enable age appropriate play/participation and sense of self-efficacy [10], which may in turn, result in a desire to practice more [11]. Completing larger volumes of training at higher intensities may allow VR training to produce greater improvements in movement and postural control [12]. A limited number of smaller studies have discussed rehabilitation utilizing virtual environments for children with CP. Three studies utilized three dimensional video-capture systems to address gross motor and reaching movements in children with CP [10,13,14]. Subjects in all three studies made improvements in motor function and measures of real-world use. The subject in the study by You et al. also demonstrated measurable changes in cortical activation associated with impaired elbow movement as measured by fMRI. Deutsch et al [15] describe a case study in which an adolescent utilized a commercially available hand-held controller to play computer games. The subject demonstrated improvements in visual perceptual processing, postural control and functional mobility at post-testing.

One of the limitations of VR for children with CP is the relatively high level of motor function required to interact with these systems [16]. One approach to broadening the group of people that can utilize VR and gaming technology for motor rehabilitation has been combining adaptive robotic systems that interface with virtual environments. These systems have been studied in the adult stroke population [17-19]

Recently, a single investigation into the use of robots for upper extremity rehabilitation for a child with CP was presented by Fasoli et al [20]. They describe a case study with a 6 year old child with upper extremity hemiplegia that performed four weeks of robotically facilitated planar reaching activities following application of botulinum toxin to reduce spasticity in elbow, wrist and finger flexors. This subject showed small improvements at the impairment level that were comparable to an equivalent volume of Occupational Therapy following botulinum toxin therapy and a corresponding increase in parent ratings of spontaneous use of the involved arm and hand.

We hypothesize that the integration of VR with robotics could be successful if applied to children with hemiplegic CP. The combined benefits of increased attention provided by VR and the large training stimulus afforded by adaptive robotics demonstrated in the stroke rehabilitation literature [18,19,21-23], may increase the beneficial effects of these two approaches synergistically. This paper will describe the design of five complex VR simulations combined with adaptive robots for the rehabilitation of upper extremity impairments and function in children with CP and examine the feasibility of this system in the context of a two subject training study.

## Methods

### Hardware

The Haptic Master^® ^(Moog, The Netherlands) combined with a ring gimbal is a 6 degree of freedom admittance-controlled (force-controlled) robot which has been used by several authors studying upper limb rehabilitation for adults with strokes [19,23].

External force exerted by the user on the robot, along with end-point position and velocity are measured in 3D in real time at a rate of up to 1000 Hz to generate reactive motion allowing the movement arm to act as an interface between the participants and the virtual environments. The ring gimbal when installed as the end effector adds the possibility of forearm rotation and records three more degrees-of-freedom. Active force that assists or resists forearm rotation (i.e., roll) is generated and recorded by the robot, the other two degrees of freedom (i.e. pitch and yaw angles) are recorded passively. The Haptic Master Application Programming Interface (API) allows us to program the robot to produce haptic effects, such as springs, dampers and constant global forces.

**Figure 1:**
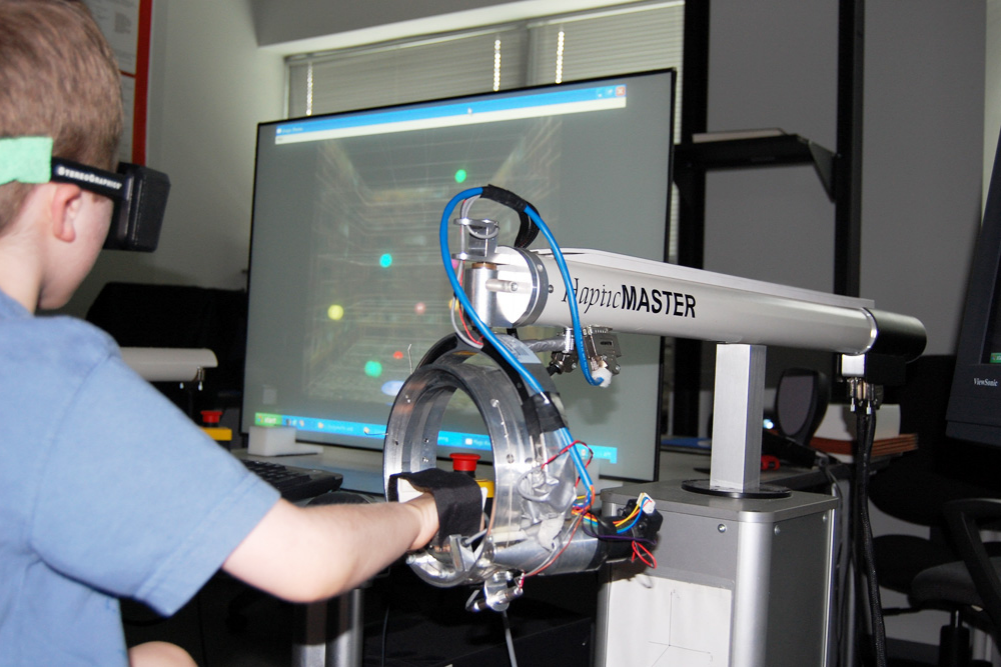
**Subject positioned in Leckey Chair interfaced with the Haptic Master using a ring gimbal**.

Three different sized forearm and hand based volar splints were fabricated to connect the subject's impaired hand to the ring gimbal. The hand based splints allow for free movement of the digits and wrists for subjects with higher levels of motor control and the forearm based splints allow free movement of the digits and provide more forearm and wrist support. Splints were chosen for each subject by their therapist in order to allow for the highest degree of freedom of movement while minimizing abnormal movement patterns. Participants were positioned in a commercially available, Advance, High Low Positioning Seat from Leckey Corporation (Ireland). The subjects in this study utilized modular foot supports, a seat belt for hip stabilization and a chest vest to prevent frontal and sagittal plane movement of the participants' trunks. The height of the Leckey Chair was oriented in relation to the HapticMaster in order to obtain a starting position of approximately 90 degree of elbow flexion with the humerus adducted to the trunk and the forearm rotated to a position of comfort according to the participant's available active forearm range of motion. Some participants in this study were not able to attain forearm neutral position due to limited range of motion (Figure 1).

### Simulations

#### Bubble explosion

**Figure 2:**
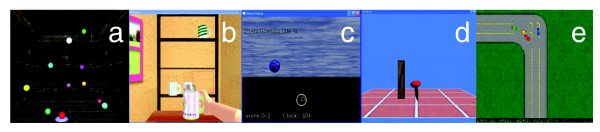
**Screen presentations of a) Bubble Explosion, b) Cup Reach, c) Falling Objects, d) Hammer, and e) Car Race**.

The Bubble Explosion simulation focuses on improving the speed and accuracy of shoulder and elbow movements during point to point reaching movements. The participant moves a virtual cursor in a 3D space in order to touch a series of ten haptically rendered bubbles with 2 cm radius, floating in the 3D environment (Figure 2a). Location of the targets is predefined in an external configuration file. In this study target placement and workspace size were standardized but they can be easily modified by therapists based on movement goals. For example, targets could be concentrated in an area of the work space that requires a combination of shoulder flexion and horizontal abduction to reach them in order to train a patient with limitations in these movements. Conversely, the entire workspace size could be reduced to accommodate a patient with a very small amount of active movement in order to allow them to interact with the simulation within their current range of abilities.

During the simulation, one of the bubble targets starts blinking when the subject's impaired hand arrives at the starting position. The subject moves the cursor toward the blinking bubble in ten seconds or less in order to make it explode. The next target bubble will start to blink when the cursor is returned to the start position. Stereoscopic glasses are used to enhance depth perception, which increases the sense of immersion and produces more normal upper extremity trajectories. Open GL stereo employs two graphic buffers, one for the left eye, another one for the right eye. Each buffer draws the same image with a different offset. The computer displays one buffer at a time with high refresh frequency (120 Hz). CrystalEyes^® ^glasses (StereoGraphics, U.S.A.), block one eye at a time with the same frequency as the computer's refresh rate. This synchronization allows the right eye to see the right graphic buffer, and the left eye to see the left graphic buffer, producing a 3-dimensional stereo effect.

#### Cup reach

The goal of the Cup Reach simulation is to improve general upper extremity strength and reaching accuracy. The screen displays a three-dimensional room with haptically rendered shelves and table. The shelves are at three different levels in height. The simulation utilizes a calibration protocol that allows the height, width and distance to the shelves to be adjusted to accommodate the active range of motion of the participant. The position of a virtual hand displayed in the simulation is controlled by the participant's hemiplegic arm. During the training, one virtual cup with handle will appear on the table, and a red square indicating the location to put the cup will be displayed on the shelf. A small target, which is a different color than the virtual hand, denotes the area of the hand used to make contact with the cup handle (Figure 2b). The participant uses their virtual hand to lift the virtual cup and place it onto the shelf. A new virtual cup will continuously appear when the previous one has been placed on the shelves until all of the nine spots have been filled. Unlike the Bubble Explosion simulation described above, in this activity, arm endpoint and viewpoint move synchronously to maintain a clear view of the virtual hand throughout reaching in order to increase the sense of involvement in the activity.

Haptic obstacles are employed in this simulation to provide feedback, which shapes trajectories performed by the participant in a similar fashion to the motor planning process used in real world environments. Collisions with the tables, shelves and other cups provide tactile feedback and actual physical task constraints which provide for feedback and feed-forward processes after the subject acclimates themselves to the virtual environment. Without haptic feedback, a participant could reach through a virtual shelf or table top. A haptically rendered version of this shelf or table will require an up and over trajectory that is closer to that required to place an object on a real world shelf [24].

The weight of the haptic cups can be adjusted, which allows for strengthening activities for less impaired participants as well as anti-gravity assisted movement for weaker participants. A damping effect can be applied by the Haptic Master, which stabilizes the subjects' arm movement in 3 dimensions.

#### Falling objects

The purpose of the Falling Objects simulation is to improve upper extremity reaching towards a moving object. Each repetition begins with the participant positioning the cursors at the starting position. As soon as the object starts falling, the participant moves the virtual cursor to catch it before it hits the ground. The higher the participant catches the object, the better score he/she will get. To train antigravity arm movement medially and laterally, objects were implemented to fall from the virtual sky either along the middle line or about 40 cm left or right of the middle line (Figure 2c). Global damping can be increased to enhance strength-training effects or stabilize the arm trajectory for participants with coordination impairments.

#### Hammer

Our original design of the Hammer simulation focuses on improving forearm pronation and supination during shoulder flexion and elbow extension in a three dimensional space. In the simulation, the position and orientation of a virtual hammer is controlled by the subject's hemiplegic arm and rotation of the forearm. During training, a target (vertically oriented wooden rod) appears in the middle of the screen, and the subject moves the hammer, which is oriented in the frontal plane, to the target and uses repetitive pronation movements to drive the target into the ground (Figure 2d). After the subjects described in this study were screened, a need to train isolated forearm supination was identified by the occupational therapist conducting the trial. The simulation was modified to allow the robot to assist the subject's impaired arm to move to a fixed location where the arm was stabilized with a strap, in order to reduce shoulder elevation and rotation, thus isolating forearm supination. Subjects rotated the forearm to control the virtual hammer to drive the target into the ground. 10 repetitive combinations of forearm pronation and supination were required to complete the task. A new target appears after each trial is completed. The rotation angle required to successfully move the hammer is adjustable for different subjects according to their impairment level. The number of targets presented and their locations can be adjusted to accommodate the participants' level of impairment and to meet the goals of the therapy. A time bar indicating the time required to complete the task appears at the end of each trial to provide participants with feedback as to how well they performed.

#### Car race

This simulation presents the subject with a track and 3 other competing cars (Figure 2e). The subject uses a slight force either forwards or backwards (perpendicular to the plane of forearm rotation) to accelerate or decelerate the car. The subject turns their car by pronating or supinating their forearm to turn the ring gimbal. A virtual spring was installed in the ring gimbal, which helps the user to return to the initial position as necessary. The stiffness and damping values of the spring can be adjusted as needed. All mechanical parameters (i.e., forearm orientation angles and magnitude of spring forces) can be modified and adjusted to adapt for different users.

The car race video game was initially obtained through open source code (The Code Project™ http://www.codeproject.com). The program was originally designed to control the car using keyboard commands. The source code was modified to accept inputs from the Haptic Master and ring gimbal to command the cars. A variety of tracks present different difficulty levels according to their shape and width and end-users can create and edit track shapes to tailor this activity to the participant's therapeutic needs. The user competes against three cars and the game allows for choosing among different difficulty levels, each level representing a different speed and competition level. The game has a sound feature to make it more exciting for the children.

### Participants

Two children, a seven year old girl (S1) and a ten year old boy (S2), both with spastic hemiplegia secondary to Cerebral Palsy (CP) were recruited from the outpatient department of a comprehensive pediatric rehabilitation facility. Children were chosen based on an ability to attend to all items on a 16 inch wide screen, demonstrate at least minimal active movement of their shoulder and elbow and tolerate at least 90 degrees of passive shoulder flexion. Pre-participation data is summarized in Table 1. All relevant information was obtained from medical records or a questionnaire completed by parents of the participants (Table 1).

**Table 1:** Subject characteristics

Subject	Age	Sex	Cognition	Impaired Hand	Dominant Hand	Ambulatory?
S1	7	F	Normal	Right	Left	No

S2	10	M	Normal	Left	Right	No

### Training procedure

Participants used the Robot Assisted Virtual Rehabilitation (RAVR) system for one hour, 3 days a week for three weeks in order to approximate a short course of outpatient therapy. Subjects performed four sets of ten reaches utilizing the Bubble Explosion simulation to initiate each session for performance testing purposes. The subjects played a combination of three or four of the other simulations depending on their therapeutic goals, tolerances and preferences for the remainder of the sixty minute session. This resulted in an average of 23 minutes of activity during the 60 minute sessions for S1 and S2. Games were modified gradually to increase difficulty in order to challenge the subjects as their performance improved. Initially subjects attempted to utilize compensatory movements to accomplish the game tasks as observed visually by therapists monitoring training. Splinting and positioning adjustments were made by the therapists to enhance typical movement patterns. In addition the starting positions and parameters (beginning AROM, resistance, and damping) on the RAVR were modified in order to physically challenge the subjects but allow for an approximate success rate of 80%. Cumulative motor fatigue was observed at varying points during training. At these points, the therapists adjusted activity parameters to prevent unintended muscle substitution patterns and to maintain approximately 50% of continuous participation for the 60 minute training session. Task parameters from the final trial of the previous session were used to initiate training for subsequent sessions.

### Measurements

Clinical testing was performed just prior to and immediately following the training period. The same licensed/registered Occupational Therapist performed both sets of clinical tests using the same equipment. Measurements included upper extremity active range of motion and strength. We measured upper extremity movement quality using the Melbourne Assessment of Unilateral Upper Limb Function (MAUULF), a sixteen activity battery designed for children with upper extremity hemiplegia [25]. Each activity is rated on a three, four or five point scale with all 16 activities summed to achieve a raw score. The raw score is divided by the total possible score to produce a percentage score [26,27]. Three of the tests included in the Melbourne Assessment including forward and lateral reaches and a hand to mouth reach were timed to assess changes in motor control and real-world upper extremity function. Kinematic measurements including hand movement speed and movement duration were calculated using data collected by the robot during the Bubble Explosion activity on the first and the last day of training as well as at the first day of each training week. Smoothness of endpoint trajectory during performance of the same activity was evaluated by integrating the third derivative of the trajectory length. This numerically describes the ability to produce smooth, coordinated, gross reaching movements versus disjointed collections of sub-movements [28,29]. Four Nest of Birds™ sensors were attached to the wrist, elbow, shoulder and trunk of the participants to measure the kinematic parameters of the impaired limb at a sampling rate of 100 Hz.

Subjects responses to the simulations were evaluated via survey and therapist report each session. Therapists determined if a subject showed fatigue during a simulation and if the subject maintained attention throughout performance of a simulation. Time to fatigue and time to break in attention was also recorded. After each simulation subjects were asked if a simulation was fun and if they would like to perform the simulation again in the future. Yes, Maybe, and No responses were recorded.

## Results

Both participants completed 9 hours of training in 3 weeks. No untoward events occurred and no adverse responses to treatment or complaints of cybersickness were reported. The games in general held the children's attention for an entire sixty minute session. Specifically, the Bubble Explosion game and the car game were more motivating to the children which allowed greater participation.

Subject S1 showed improvements in their overall performance on the Melbourne Assessment (Table 2), with the overall percentage score increasing from 59.8 to 67.2. She demonstrated improvement on all of the MAUULF items involving upper extremity elevation except hair combing, which correlates with her improvements on the three timed components of the Melbourne Assessment (Table 2). She also improved in the" hand to mouth and down" item but did not improve on the pronation-supination item despite her improvement in supination AROM. Subject S2 did not demonstrate improvements in the "Forward..." or "Sideways Reaching to an Elevated Position" items from the MAUULF despite improvements in speed during these movements. He scored higher initially than S1 on these items possibly suggesting a ceiling effect on sensitivity."Reaching to opposite shoulder" performance improved, as did "hand to mouth and down "performance. His MAUULF pronation-supination score did not change, despite a large improvement in supination AROM. S2 only improved 0.9 percent on his MAUULF composite score but made substantial improvements in active range of motion (Table 3) and kinematic measures of his performance on the Bubble Explosion reaching activity (Table 4). S2 achieved a 15 degree increase in active shoulder flexion (from 130 to 145), and a 50 degree increase on forearm supination (from -60 to -10). No standards for clinically significant change as they relate to active range of motion measurements in this population have been established, but the impact of range of motion impairments on function in children with CP is supported by the rehabilitation literature [29,30].

**Table 2:** Upper extremity function testing

	MAUULF %		Forward Reach Time (s)		Reach sideways Time (s)		Hand to Mouth Time(s)	
	**Pre**	**Post**	**Pre**	**Post**	**Pre**	**Post**	**Pre**	**Post**

S1	59.8%	67.2%	2.9	1.5	2.2	0.8	5.4	4.6

S2	76.2%	77.1%	4.5	1.5	2.4	1.8	2.2	1.6

**Table 3:** Impairment measurements

Subject	Strength						Active Range of Motion					
	**Grip**		**Lateral Pinch**		**3-Jaw Pinch**		**Shoulder Flexion**		**Elbow Flexion**		**Supination**	

	**pre**	**post**	**pre**	**post**	**pre**	**post**	**pre**	**post**	**pre**	**post**	**pre**	**post**

S1	6	14	3	7	1	2	150	145	140	140	0	0

S2	3	3	2	4	1	2	130	145	140	140	-60	-10

**Table 4:** Percent change in reaching kinematics

	Duration	Path Length	Smoothness
S1	0.94%	18.02%	-0.99%

S2	68%	64%	92%

Both S1 and S2 had an almost 100% increase on strength tests. S1's grip strength increased from 6 lbs to 14 lbs, lateral pinch strength increased from 3 lbs to 7 lbs, and 3-jaw pinch strength increased from 1 lb to 2 lbs. S2's lateral pinch strength increased from 2 lbs to 4 lbs, and 3-jaw pinch strength increased from 1 lb to 2 lbs. These gains are interesting based on the fact that grip and hand strength were not specifically trained during the intervention. Similar improvements of smaller magnitude in distal function in response to proximal upper extremity robotic training have been described in the adult stroke literature [31].

**Figure 3:**
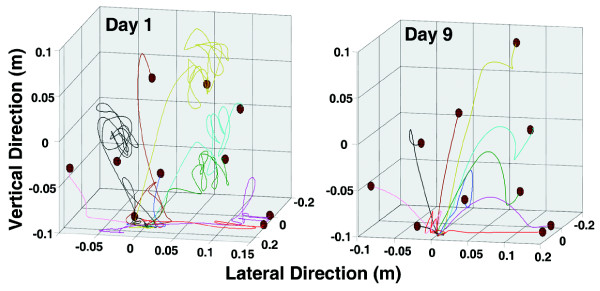
**Right panel) Hand trajectories performed to accomplish the Bubble Explosion simulation task on day one by subject S2**. Left Panel) Hand trajectories of the same subject performing the Bubble Explosion task on the final day of training.

**Figure 4:**
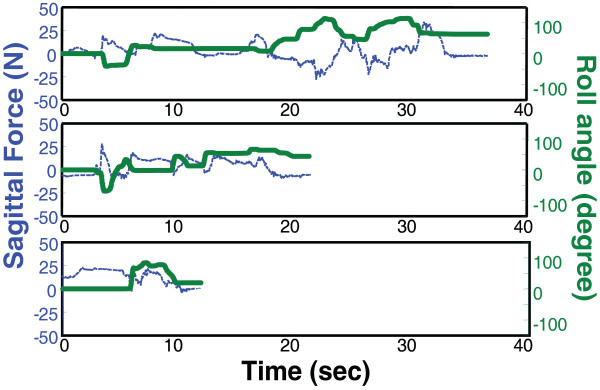
**Depicts subject S2 making a single right turn during the Car Race simulation, on three separate occasions over the training period**. Green bold line depicts roll angle. Blue thin line is horizontal (pushing) force. S2's ability to coordinate the sagittal plane pushing needed to accelerate the car with the supination required to turn the car progresses from multiple unsuccessful attempts on day one (top panel), to a slow and disjointed sweeping turn on day five(middle panel), to a single sharp turn without a loss in speed on day 9 (bottom panel).

Both participants showed improvement on several kinematic measures of the movement recorded directly by the robot, during the Bubble Explosion activity. Figure 3 demonstrates the hand trajectories performed to accomplish this task on day one and day nine by subject S2. Trajectories became more accurate and stable. The percentage of improvement between pre-test and post-test for several kinematic measures including smoothness, a measurement of the ability to perform a single well-integrated movement, and two measures of efficiency (path length and duration) are shown in Table 4. The improvements in stability and accuracy demonstrated by S2 in Figure 3 are supported by improvements in these analyses (Table 4). S1 made similar improvements between day 1 and 6 but failed to maintain them over the entire length of the study period. S1 began school after her sixth training day and was unable to perform at the level she previously achieved following a full day of school. Figure 4 tracks the progress of S2 over the training period making a single right turn during the Car Race simulation. S2's ability to coordinate the sagittal plane pushing needed to accelerate the car with the supination required to turn the car progresses from multiple unsuccessful attempts on day one, to a slow and disjointed sweeping turn on day five, to a single sharp turn without a loss in speed on day 9.

Subject response data for two of the simulations proved to be interesting. Hammer and Car Race both train supination, an area of impairment for both subjects, but subject response to the two simulations differed. Both subjects performed Hammer simulation 4 times. S1 demonstrated decreased attention in 2 of the 4 sessions with this simulation and fatigue in 3 of the 4 sessions. S2 demonstrated decreased attention during three of his 4 sessions and fatigue during 4 of his sessions performing the Hammer simulation. Neither subject described the activity as fun and never agreed to perform the simulation again in the future. However, both subjects agreed to try the simulation again during subsequent sessions and both subjects demonstrated gradual increases in tolerance for the activity. In contrast, the Car Race simulation proved to be the most popular simulation with no attention lapses, no demonstrations of fatigue and unanimous agreement that the simulation was fun and an option for future sessions. The other simulations did not display a consistent response pattern.

### Limitations of the system and current study

The graphics and game action featured in our simulations is rudimentary in comparison to commercially available games. Future iterations of our simulations targeted for children will be designed by computer engineers with gaming industry backgrounds in collaboration with our team of biomedical engineers in an attempt to bridge this gap.

Because of the higher levels of functioning of both of our subjects, this study did not fully test the feasibility of the system's robotic assistance capabilities. Future studies with lower functioning children are indicated. An important addition to our outcome battery should be a measure of changes in activities of daily living.

## Discussion

This study establishes the feasibility of the NJIT-RAVR system for use by young children with mild to moderate hemiplegia secondary to CP. Both subjects completed 9 hours of training without ill effects. Both subjects demonstrated improvement in kinematic and performance measures as collected by the robotic system. S1 made improvements in coordination and efficiency of movement as evidenced by the timed elements of the Melbourne Assessment. S2's changes at the functional level as measured by the Melbourne Assessment were small, but he made substantial improvements in active range of motion at the shoulder and elbow. It is possible that this subject may require more time to integrate these expanded motor abilities into improvements in function. Another possible explanation is that S2's pretest scores were high in many of the MAUULF domains trained during this intervention, making MAUULF composite improvements less likely.

One aspect of the system described in this paper is its flexibility. The Hammer task was modified from its original iteration to specifically address the therapeutic goals identified by S2's therapy team. One of S2's most significant impairments was decreased active supination, a common impairment for children with hemiplegic CP. Under the direction of S2's therapist, the Hammer task parameters were modified to train supination with his elbow fixed at 90 degrees of flexion. This flexibility allowed S2's training to address this impairment. During the three-week training period S2 gained approximately 50 degrees of active supination.

Two of the five simulations discussed in this paper were originally designed for the rehabilitation of adults. One simulation required modification to maintain interest in our younger subjects. In the original Bubble Explosion simulation, bubbles simply disappeared when the virtual cursor reached them. Children lost interest quickly. In order to maintain attention to this task, an explosion scene and an option to select the sound heard when bubbles explode was added. Generic cartoon, animation, animal and Halloween sounds were included in the sound effect options to create a more "game-like" environment. This resulted in increased time on task for both subjects.

The volume of sensory stimulation provided by a virtual environment, when used for the rehabilitation of people with neurological impairments, needs to be considered. Some authors working with adults after strokes endeavor to keep their visual presentations simple [22] and others grade the visual and auditory presentations to accommodate varying levels of processing ability [17]. The interaction between the ability to process sensory stimuli and the ability to span attention in children with CP has not been established and developing methods to assess the optimal volume of sensory stimuli for a patient will require further study.

The simulations described in this paper were constructed using a variety of design approaches. Source code for Car Race and Falling Objects was obtained from the Internet and adapted to utilize inputs from the Haptic Master as game controls. Bubble Explosion and Hammer were designed as original programs in C++/OpenGL. Each of the approaches utilized offer advantages and disadvantages but all should be considered by scientists and commercial interests in the process of expanding this area of rehabilitation research.

The combination of adaptive robotics and game-like virtual environments offers promise in the ability of both approaches to expand the volume and intensity of practice a participant can perform [18,19]. Neither of the subjects in this study demonstrated problems with performing more than 25 minutes of active training during a 60 minute session using our system. The two subjects involved in this study were capable of exerting against gravity movement of their upper extremities. Previous iterations of the RAVR system tested on subjects with strokes were designed to assist subjects that were unable to generate sufficient muscular force to complete a movement against gravity. The system allows the participant to initiate and execute as much of a movement as they are able and then assists them allowing the subject to experience a degree of success while it forces them to work at the highest level they are capable of. Adaptive robotics may allow lower functioning children to access the expanded attention to task afforded by VR as well. At a point in training at which children would fatigue physically and have their performance decay, assistance levels provided by the robot could increase, allowing them to complete the number of repetitions necessary without undue fatigue. Expanding training times beyond the sixty minutes performed in this study will be an area for future study. Another will be to investigate the use of this system on a sample of children with a wider range of impairments.

## Consent

Written informed consent was obtained from each subjects parent's for publication of this case report and accompanying images. A copy of the written consent is available for review by the Editor-in-Chief of this journal.

## Competing interests

The authors declare that they have no competing interests.

## Authors' contributions

QQ participated in the robotic/VR system design, data collection, data analysis initial manuscript preparation and revision. DAR participated in the robotic/VR system design, data collection, data analysis, initial manuscript preparation and revision. SS participated in the robotic/VR system design, data collection, data analysis, initial manuscript preparation and revision. GGF participated in data analysis, initial manuscript preparation and manuscript revision. DK participated in the study design, subject recruitment, data collection and manuscript revision processes. HDP participated in the study design, subject recruitment, data collection and manuscript revision processes. SVA participated in the robotic/VR system design, study design, data analysis and manuscript revision processes. All authors read and approved the final manuscript.
